# Fault Diagnosis Method of Planetary Gearboxes Based on Multi-Scale Wavelet Packet Energy Entropy and Extreme Learning Machine

**DOI:** 10.3390/e27080782

**Published:** 2025-07-24

**Authors:** Rui Meng, Junpeng Zhang, Ming Chen, Liangliang Chen

**Affiliations:** 1 School of Mechanical Engineering, Anhui University of Technology, Maanshan 243002, China; 15257590183@163.com; 2 AHUT Engineering Research Institute, Anhui University of Technology, Maanshan 243002, China; dxx_0327@163.com (J.Z.); 18360719853@163.com (M.C.)

**Keywords:** planetary gearbox, gear fault diagnosis, multi-scale wavelet packet energy entropy, ELM

## Abstract

As critical components of planetary gearboxes, gears directly affect mechanical system reliability when faults occur. Traditional feature extraction methods exhibit limitations in accurately identifying fault characteristics and achieving satisfactory diagnostic accuracy. This research is concerned with the gear of the planetary gearbox and proposes a new approach termed multi-scale wavelet packet energy entropy (MSWPEE) for extracting gear fault features. The signal is split into sub-signals at three different scale factors. Following decomposition and reconstruction using the wavelet packet algorithm, the wavelet packet energy entropy for each node is computed under different operating conditions. A feature vector is formed by combining the wavelet packet energy entropy at different scale factors. Furthermore, this study proposes a method combining multi-scale wavelet packet energy entropy with extreme learning machine (MSWPEE-ELM). The experimental findings validate the precision of this approach in extracting features and diagnosing faults in sun gears with varying degrees of tooth breakage severity.

## 1. Introduction

Due to their advantages of simple structure, small volume, large transmission ratio, and small power loss [1], planetary gearboxes have been widely used in automotive, aviation, generator, wind turbine, crane, and other fields [2]. However, in these fields, planetary gearboxes frequently operate under demanding conditions, including high speeds, heavy loads., and unstable conditions, which can easily cause gear faults such as broken teeth and wear cracks [3]. Among the various fault types in planetary gearboxes, gear tooth breakage faults account for a significant percentage of the failure modes encountered in planetary gearboxes. This is mainly due to long-term overloading operation, which causes gears to bear excessive torque and thus leads to breakage. This kind of malfunction has a crucial impact on the reliability and performance of the planetary gearbox, resulting in a decrease in transmission efficiency and even possibly leading to the failure of the mechanical device [4]. At present, much substantial progress has been achieved in investigations of the dynamic characteristics of gear tooth breakage, cracks, and wear faults. However, most of the studies are limited to the identification of gear fault types, and there are relatively few studies on the degree of gear faults. Therefore, for the problem of analyzing the degree of gear tooth breakage faults in planetary gear trains, a study of the fault vibration characteristics in the planetary gearbox system, revealing their variation patterns and performing fault diagnosis, is essential. Studies on planetary gearbox broken tooth faults will provide theoretical support for the assessment of tooth damage severity and contribute to the accurate diagnosis of faults in planetary gear systems, which is of critical importance for enhancing the reliability of gearboxes and guiding maintenance.

Regarding the extraction and recognition classification of fault features, researchers have proposed various theories, including wavelet transform (WT), empirical mode decomposition (EMD) [5], local mean decomposition (LMD), empirical wavelet transform (EWT), and variational mode decomposition (VMD) [6]. Time–frequency analysis methods effectively decompose and characterize the response signals of early bearing faults. Wavelet transform (WT) is an efficient approach for time–frequency analysis, known for its strong noise reduction capability [7]. Wavelet packet transform (WPT), an enhancement of WT, decomposes signals at multiple scales with higher resolution based on their frequency distribution [8]. Its core value lies in breaking through the limitations of traditional wavelet decomposition in processing high-frequency bands, providing a more powerful tool for high-frequency transient feature extraction, multi-scale pattern recognition, and noise robustness analysis. This leads to more uniform frequency feature extraction, enabling the uniform extraction of features from different frequency bands of fault impact signals and facilitating the intelligent classification of various signal groups. Fan et al. [9] developed a wavelet-based statistical signal detection technique for incipient composite fault monitoring and diagnosis in bearings. Bin et al. [10] integrated wavelet packet decomposition and empirical mode decomposition to isolate characteristic fault frequencies to facilitate the early detection of faults in rotating machinery. Wang et al. [11] introduced a novel methodology for rolling bearing diagnosis, integrating wavelet packet decomposition with sparse representation theory to leverage sparse wavelet reconstruction residual features. Wan et al. [12] substituted binary wavelet packet transform for finite impulse response filter banks as the band segmentation technique to optimize the fast spectral kurtosis algorithm. Chen et al. [13] constructed feature vectors characterizing different rolling bearing fault types by computing the energy entropy values of sub-signals derived from WPT decomposition across distinct frequency bands. However, in the case of multiple fault signal combinations in their research, it is difficult to accurately and effectively extract the fault features and correctly identify the fault types.

Recently, various entropy-based complexity metrics based on information theory have been introduced for feature extraction from nonlinear vibration signals [14]. Entropy is a fitting metric for evaluating the regularization process and the underlying complexity of data sequences. As a measure of uncertainty and irregularity within temporal sequences [15], it has been extensively employed for extraction due to its advantages of good clustering ability and classification accuracy of the dynamic characteristics of rotating machinery [16]. Examples include approximate entropy (ApEn) [17], sample entropy (SampEn) [18], multi-scale entropy (MSE) [19], fuzzy entropy [20], etc. Li et al. [21] proposed a new fault feature extraction method for rolling bearings that consists of ensemble empirical mode decomposition (EEMD) and improved band entropy (IFBE), aimed at capturing early weak fault features of rolling bearings. Li et al. [22] proposed a signal processing method for planetary gearboxes under non-stationary conditions that combines an improved Vold–Kalman filter with multi-scale sample entropy, which has a good effect on fault type identification in planetary gearboxes. Zheng et al. [23] proposed a fine composite multi-scale fuzzy entropy method based on sigmoid and applied it to the dynamic complexity analysis of mechanical vibration. Sun et al. [24] proposed a multi-scale fractional wavelet packet energy entropy and synchronous optimization strategy. They combined wavelet packet decomposition with the information entropy theory and introduced it into energy calculations. The coarse-grained theory and fractional calculus formed the multi-scale fractional wavelet packet decomposition energy entropy (FWPDE) and combined the binary particle swarm optimization feature set and the synchronous optimization strategy of SVM parameters. The contactless fault characteristics of the sound signal of the railway turnout machine were used, and the accuracy of the method was verified by comparing the conclusions. Rodriguez et al. [25] proposed the stationary wavelet dispersion entropy and the stationary wavelet permutation entropy and combined the KELM with the Gaussian kernel function to replace the activation function of the extreme learning machine for the fault diagnosis of rolling bearings. By comparing it with the stationary wavelet singular value entropy, it was verified that this method has a better diagnostic effect on rolling bearings with different fault types. Some of these studies lack experiments to verify their effectiveness under different types and severity of faults, and there are relatively few studies on the degree of damage caused by mechanical equipment failures.

With artificial intelligence technology having advanced and evolved, the primary applications in fault diagnosis lie in methods based on machine learning and deep learning for training and learning feature information, delivering diagnostic results in terms of fault classification. Intelligent diagnostic systems are typically built by training models based on extracted fault features. Commonly used methods include support vector machines (SVMs), convolutional neural networks (CNNs), Long Short-Term Memory (LSTM), etc. CNN was proposed by LeCun [26] and utilizes operations such as convolution, nonlinear transformation, and downsampling to process known grid-like input data such as two-dimensional images or one-dimensional time series. He et al. [27] proposed a mechanical intelligent fault diagnosis method based on the Ensemble Multi-scale Convolutional Attention Network (EMCAN) to solve the problems of poor anti-noise performance and easy neglect of minority class samples in traditional convolutional neural networks and verified the effectiveness and superiority of this method. LSTM deep learning has long-term memory ability, which can effectively solve the problems of long-term dependence and explosion gradient caused by excessively long sequences. Zhu et al. [28] constructed a fault diagnosis model with reliability and generalization ability by using LSTM combined with transfer learning that introduces L1 regularization. Extreme learning machine (ELM) is a novel machine learning approach, categorized as a supervised learning algorithm. It is primarily employed for classification and regression tasks. ELM demonstrates significant advantages, including rapid learning speed and strong generalization capabilities, making it particularly suited for handling large-scale datasets [29]. It has been extensively utilized in fault diagnosis. ELM optimizes an iterative process and establishes fault diagnosis models by selecting appropriate adjustable parameters associated with the input and hidden layers [30]. Tian et al. [31] incorporated the singular value vector as a representative fault feature within the ELM framework to automate bearing fault classification, expediting the diagnostic speed. Mao et al. [32] addressed the issue that actual bearing fault data is unbalanced and much less numerous than normal data and proposed an online sequential prediction and diagnosis method specifically tailored for such unbalanced fault scenarios based on ELM. Cheng et al. [33] used a particle swarm optimization algorithm to determine the optimal input weights and hidden layer neuron thresholds for ELM, thereby enhancing the accuracy of diagnostic outcomes. However, many algorithms demonstrate weak local search performance and a slow rate of convergence when tackling complex problems [34].

Based on the above analysis, this study addresses several key issues in the fault diagnosis of planetary gear systems in gearboxes: the difficulty in extracting fault information and local features associated with varying degrees of tooth breakage in the sun gear; the scarcity of research specifically targeting diagnosis methods for tooth breakage fault severity in gearboxes; the inability of traditional entropy-based analysis methods to fully reflect fault information when processing raw vibration signals; and low diagnostic accuracy. The central purpose of this paper is to resolve the above-mentioned issues by proposing a gear fault feature extraction method based on multi-scale wavelet packet energy entropy. Energy entropy measures the energy distribution and complexity of the signal, enabling it to effectively identify the energy changes in the vibration signals caused by gear faults, capture the uneven distribution of energy and the spectral changes resulting from broken teeth, and reflect the change in the degree of broken teeth through the concentration degree of energy, thereby revealing the abnormal features in the signal. However, it is insensitive to low-energy faults and does not consider the temporal relationship. By introducing the coarsening method of multi-scale entropy and combining it with wavelet packet theory to form multi-scale wavelet packet energy entropy, it can effectively address the shortcomings of energy entropy in fault diagnosis and more accurately extract fault features, which is mainly used for identifying the degree of broken teeth of the sun gear and fault diagnosis in the planetary gearbox system. This method enables the extraction of characteristic information across various frequency ranges, fully reflects fault information, and conducts multi-scale analysis. It effectively splits and extracts comprehensive gear fault signal features. Furthermore, it combines fault diagnosis algorithms based on artificial intelligence to accurately diagnose and classify gear faults, thereby improving the accuracy of fault diagnosis. This study provides strong support for subsequent fault diagnostics and offers valuable references for the maintenance and management of gearboxes.

The main innovation points and research contents of this paper are as follows:

(1) A fault diagnosis approach utilizing multi-scale wavelet packet energy entropy is proposed. The wavelet packet algorithm is employed to decompose and reconstruct vibration signals at multiple scales and directions. The wavelet packet energy entropy of nodes with different scale factors under various operating conditions is calculated to form the signal’s feature vector, thereby accurately extracting the fault characteristics of vibration signals corresponding to different tooth fractures of the sun gear.
(2) A gear fault diagnosis method with high computational efficiency and accurate diagnostic results is proposed by combining the multi-scale wavelet packet energy entropy feature extraction method with the ELM(MSWPEE-ELM) diagnosis model. The diagnostic results validate the practicality and high efficiency of the method.
(3) The MSWPEE-ELM fault diagnosis is compared with other methods to verify the superiority and feasibility of the proposed scheme. The results show that this method has high accuracy and prove the efficiency of the proposed method in the fault diagnosis of different degrees of broken teeth of the solar gear.

The contents of each section are distributed as follows: Section 2 presents the theoretical foundation, including the improved multi-scale wavelet packet energy entropy theory and the calculation model of the extreme learning machine. Section 3 outlines the operational steps and fault diagnosis flow chart of the MSWPEE-ELM gear tooth fracture fault diagnosis method, and introduces the relevant information of the experimental setup. Section 4 provides an experimental analysis of the vibration signals caused by different tooth fracture faults in the sun gear of the planetary gearbox system and the proposed method is compared with several other fault diagnosis methods, validating the feasibility and high efficiency of the proposed method. In Section 5, the paper concludes with a summary of the research and a discussion of the issues and future directions of the study.

## 2. Basic Theory

In practical applications, traditional fault diagnosis methods such as the empirical mode decomposition (EMD) algorithm [35], when processing vibration signals in the presence of noise, can lead to the generation of redundant Intrinsic Mode Functions (IMFs) and cause uncertainty in the decomposition sequence [36]. To handle nonlinear and non-stationary signals, wavelet packet analysis can be employed for adaptive decomposition by selecting different wavelet functions and adjusting the number of decomposition layers. This method offers the advantage of shorter computation time and does not require correlation coefficients for filtering IMF components. However, wavelet packet analysis is associated with the issue of uneven decomposition resolution. Therefore, selecting appropriate parameters for the wavelet basis and decomposition layers is crucial. Furthermore, it fails to comprehensively detect and extract local features from the original vibration signals. Additionally, when using energy entropy for diagnosing gear faults, challenges arise, including the difficulty of accurately identifying fault features and low diagnostic accuracy. To address this issue, this section presents a novel approach based on multi-scale wavelet packet energy entropy in gear fault classification, substantiating the advantages of this approach in improving classification accuracy and indicating fault types.

### 2.1. Wavelet Packet Theory

Wavelet packet analysis is an extension of wavelet theory that decomposes a signal into multiple sub-frequency bands [37]. In this analysis, the signal is decomposed into a series of wavelet packet basis functions, each corresponding to a specific frequency range and time period. These basis functions are constructed by applying different decompositions and filters to the parent wavelet function. During the decomposition process, the signal is decomposed into multiple sub-frequency bands. The frequency range and time period of each sub-frequency band are related to the wavelet packet basis function. Compared with traditional wavelet analysis, wavelet packet analysis offers higher frequency resolution and is better suited for handling non-stationary signals. These advantages ensure the accuracy of wavelet packet transforms in gear fault detection and identification research.

The wavelet packet decomposition process of the discrete time sequence *x*(*n*) is as follows [38,39]:

(1) Initial condition

(1) $$w_{0}^{0}(n)=x(n)$$

where $w_{j}^{k}\left(n\right)$ represents the discrete coefficient sequence of the *k*-th sub-band at the *j*-th layer $\left(j\geq 0,k=0,1,\ldots ,2^{j}-1\right)$ . At the initial stage (*j* = 0), there is only one sub-band (*k* = 0), which is the original signal itself.

(2) Decomposition at the first layer

The original signal is decomposed into low-frequency and high-frequency sub-bands.

Low-frequency sub-band (*k* = 0):

(2) $$w_{1}^{0}(n)=\sum_{m\in Z}w_{0}^{0}(m)\cdot h(2n-m)$$

High-frequency sub-band (*k* = 1):

(3) $$w_{1}^{1}(n)=\sum_{m\in Z}w_{0}^{0}(m)\cdot g(2n-m)$$

where h(n) is the low-pass filter and g(n) is the high-pass filter, satisfying the orthogonal duality relationship: $g(n)={\left(-1\right)}^{1-n}h(1-n)$ .

(3) Decomposition at the *j*-th layer

For any layer *j*, the sub-band of the (*j* − 1)-th layer is decomposed into two sub-bands of the *j*-th layer:

Low-frequency sub-band (2*k*):

(4) $$w_{j}^{2k}(n)=\sum_{m\in Z}w_{j-1}^{k}(m)\cdot h(2n-m)$$

High-frequency sub-band (2*k* + 1):

(5) $$w_{j}^{2k+1}(n)=\sum_{m\in Z}w_{j-1}^{k}(m)\cdot g(2n-m)$$

The decomposition of each layer is based on the sub-band coefficients of the previous layer implemented through the same low-pass/high-pass filtering and downsampling operations. In addition, the $2^{j}$ sub-bands at the *j*-th layer correspond to equal-width frequency intervals that cover the entire frequency band of the original signal (from *DC* to the Nyquist frequency).

**Figure 1:**
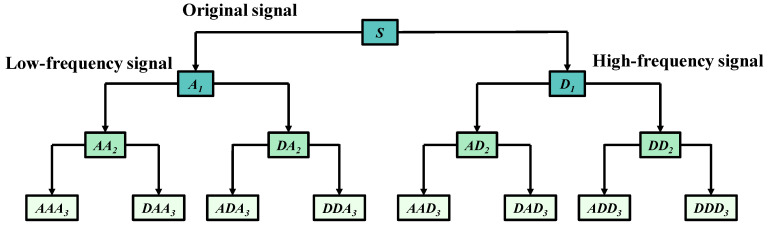
The three-layer decomposition tree diagram of the wavelet packet.

According to Figure 1, the wavelet packet transform splits the original signal into three layers, improving the resolution of the signal, especially by decomposing the high-frequency part of the signal layer by layer. Among them, *S*, *A*, and *D,* respectively, represent the original signal, the low-frequency signal, and the high-frequency signal. The expression of the original signal after wavelet packet decomposition is:

(6) $$S=AAA_{3}+DAA_{3}+ADA_{3}+DDA_{3}+AAD_{3}+DAD_{3}+ADD_{3}+DDD_{3}$$

After wavelet packet transformation, it can be observed that each node contains relatively independent features, and these features are composed of the information contained in each node.

### 2.2. Multi-Scale Wavelet Packet Energy Entropy

To examine the signal properties in the frequency and time domains in a comprehensive manner and to assess the uniformity of the system’s energy distribution, energy entropy is introduced as a method for entropy analysis. Energy entropy is employed to describe the uniformity or non-uniformity of energy distribution in signals or systems and to assess the complexity or regularity of time series constrained to a single scale. However, studies have shown that there is no direct correspondence between the dynamic complexity of time series and their entropy value. (During the operation of the gear system, due to wear, broken teeth, or other faults, different vibration patterns are usually generated. These vibration signals can be regarded as time series data.) Although the entropy value increases with the degree of disorder, a higher entropy value does not necessarily indicate an increase in dynamic complexity [40]. For instance, the entropy value of a time series after random processing may be higher than that of the original series, but it will destroy the inherent correlation and reduce the informational content. Additionally, the entropy of 1/*f* noise can be lower than that of white noise; however, this does not necessarily indicate that white noise possesses lower complexity than 1/*f* noise. Therefore, solely considering the entropy value at a single scale is insufficient; it is essential to examine the information of the time series at multiple scales. To solve this problem, Costa et al. added scale factors based on energy entropy and proposed the concept of multi-scale entropy (MSE) [19,41,42]. MSE analyzes time series across multiple scales through coarse graining. It considers the entropy at a single scale by incorporating the information from other scales of the time series. The entropy value and complexity of rotating machinery vibration signals exhibit variations corresponding to distinct fault types. Each fault typically manifests within a specific frequency band, and each fault has a distinct characteristic frequency band. When a fault occurs, alterations will manifest in both the signal complexity and the vibrational characteristics specifically.

Therefore, merely considering the energy entropy characteristics of the vibration signal cannot fully reflect the fault information. Multi-scale analysis is required. This section draws on the coarse-grained method of multi-scale entropy (MSE) [43,44] and, in combination with the definition of energy entropy, proposes the concept of multi-scale wavelet packet energy entropy (MSWPEE). The calculation steps of multi-scale wavelet packet energy entropy are as follows:

(1) Suppose the signal sequence $X=\left\{x_{1},x_{2},\ldots ,x_{n}\right\}$ to be analyzed has a length of N . Under different scale factors, a new coarse-grained sequence $Y\left(\tau \right)=\left\{y_{1},y_{2},\ldots ,y_{j},\ldots ,y_{\left[\frac{N}{\tau }\right]}\right\}$ is established:

(7) $$y_{j}=\frac{1}{\tau }\sum_{i=(j-1)\tau +1}^{j\tau }x_{i},1\leq j\leq [N/\tau ]$$

where τ is a positive integer and is called the scale factor. When τ=1 , the coarse-grained sequence $Y\left(1\right)$ is the original sequence. For non-zero τ , the original sequence X generates a coarse-grained sequence Y of length $\left[\frac{N}{\tau }\right]$ (indicating an integer no greater than $\frac{N}{\tau }$ ).

(2) The signal is split into the j -layer wavelet packet. After the wavelet packet decomposition, $2^{j}$ sub-signals of different frequency bands are obtained.
(3) The wavelet packet reconstruction is performed on each frequency band of the decomposed sub-signals, and the reconstruction coefficient is represented as $S_{j,k}\left(k=0,1,2,\ldots ,2^{j}-1\right)$ .
(4) Calculate the energy value $E_{j,k}$ of each frequency band:

(8) $$E_{j,k}={\int_{t_{i-1}}^{t_{i}}\left|A_{i}\left(t\right)\right|}^{2}dt$$

where $A_{i}(t)$ represents the amplitude value at time *t*; $t_{i-1}$ and $t_{i}$ are, respectively, the signal’s start time and finish time at this node.

(5) Calculate the total energy value E :
(9) $$E=\left(\sum_{k=0}^{2^{j}-1}{\left|E_{j,k}\right|}^{\frac{1}{2}}\right)$$
(6) Calculate the proportion $P_{j,k}$ of the energy of each child node to the total energy:
(10) $$p_{j,k}=\frac{E_{j,k}}{E}$$
(7) Calculate the energy entropy value of the k wavelet packet in the j layer after signal decomposition:
(11) $$H_{j,k}=-\sum_{i=1}^{N}p_{j,k}\left(i\right)\ln\left|p_{j,k}\left(i\right)\right|$$

### 2.3. Extreme Learning Machine

The extreme learning machine (ELM) is a novel machine learning algorithm widely used in the processing of regression and classification problems [45]. ELM is a machine learning algorithm similar to the single hidden layer feedforward neural network (SLFN) model. Unlike SLFN, ELM replaces gradient-based algorithms with the random initialization of input layer weights and biases. ELM utilizes generalized inverse matrix theory to solve the connection weights, optimizing the iterative training process typically required by traditional neural networks, thus significantly improving training speed. The implementation of ELM is relatively simple, requiring only the determination of the quantity of hidden layer nodes and the selection of an appropriate activation function. When dealing with a large number of training samples, ELM demonstrates strong generalization performance, effectively addressing regression and classification tasks [46].

ELM consists of the input layer, hidden layer, and output layer. The neurons in the input layer and the hidden layer, as well as the hidden layer and the output layer, are fully connected. Among them, there are *n* neurons in the input layer, corresponding to *n* input variables. There are *l* neurons in the hidden layer. The output layer has *m* neurons, corresponding to *m* output variables. Suppose the connection weights *w* between the input layer and the hidden layer are as follows:

(12) $$w={\left[\begin{array}{cccc} w_{11} & w_{12} & \ldots & w_{1n}\\ w_{21} & w_{22} & \ldots & w_{2n}\\ \vdots & \vdots & & \vdots \\ w_{l1} & w_{l2} & \ldots & w_{ln} \end{array}\right]}_{l\times n}$$

where $w_{ji}$ represents the connection weight between the *i*-th neuron in the input layer and the *j*-th neuron in the hidden layer.

Suppose the connection weight μ between the hidden layer and the output layer is as follows:

(13) $$\mu ={\left[\begin{array}{cccc} \mu_{11} & \mu_{12} & \ldots & \mu_{1m}\\ \mu_{21} & \mu_{22} & \ldots & \mu_{2m}\\ \vdots & \vdots & & \vdots \\ \mu_{l1} & \mu_{l2} & \ldots & \mu_{lm} \end{array}\right]}_{l\times m}$$

where $\mu_{jk}$ represents the connection weight between the *j*-th neuron in the hidden layer and the *k*-th neuron in the output layer.

Suppose the threshold *b* of the neurons in the hidden layer is as follows:

(14) $$b={\left[\begin{array}{c} b_{1}\\ b_{2}\\ \vdots \\ b_{l} \end{array}\right]}_{l\times 1}$$

Suppose the input matrix *X* and the output matrix *Y* of the training set with *Q* samples are, respectively:

(15) $$X={\left[\begin{array}{cccc} x_{11} & x_{12} & \ldots & x_{1Q}\\ x_{21} & x_{22} & \ldots & x_{2Q}\\ \vdots & \vdots & & \vdots \\ x_{n1} & x_{n2} & \ldots & x_{nQ} \end{array}\right]}_{n\times Q}$$

(16) $$Y={\left[\begin{array}{cccc} y_{11} & y_{12} & \ldots & y_{1Q}\\ y_{21} & y_{22} & \ldots & y_{2Q}\\ \vdots & \vdots & & \vdots \\ y_{m1} & y_{m2} & \ldots & y_{mQ} \end{array}\right]}_{m\times Q}$$

Suppose the activation function of the neurons in the hidden layer is g(x) ; then, the output *T* of the network is

(17) $$T={\left[\begin{array}{cccc} t_{1}, & t_{2}, & \ldots , & t_{Q} \end{array}\right]}_{m\times Q}$$

(18) $$t_{j}={\left[\begin{array}{c} t_{1j}\\ t_{2j}\\ \vdots \\ t_{mj} \end{array}\right]}_{m\times 1}={\left[\begin{array}{c} \sum_{i=1}^{l}\mu_{i1}g(w_{i}x_{j}+b_{i})\\ \sum_{i=1}^{l}\mu_{i2}g(w_{i}x_{j}+b_{i})\\ \vdots \\ \sum_{i=1}^{l}\mu_{im}g(w_{i}x_{j}+b_{i}) \end{array}\right]}_{m\times 1}(j=1,2,\ldots ,Q)$$

where $w_{i}=\left[\begin{array}{cccc} w_{i1}, & w_{i2}, & \ldots , & w_{in} \end{array}\right]$ ; $x_{j}={\left[\begin{array}{cccc} x_{1j}, & x_{2j}, & \ldots , & x_{nj} \end{array}\right]}^{T}$ .

Equations (17) and (18) can be expressed as

(19) $$H\mu =T^{\prime }$$

where $T^{\prime }$ is the transpose of matrix *T*; *H* is called the output matrix of the hidden layer of the neural network, and its specific form is as follows:

(20) $$H(w_{1},w_{2},\ldots ,w_{l},b_{1},b_{2},\ldots ,b_{l},x_{1},x_{2},\ldots ,x_{Q})={\left[\begin{array}{c} g(w_{1}\cdot x_{1}+b_{1})g(w_{2}\cdot x_{1}+b_{2})\ldots g(w_{l}\cdot x_{1}+b_{l})\\ g(w_{1}\cdot x_{2}+b_{1})g(w_{2}\cdot x_{2}+b_{2})\ldots g(w_{l}\cdot x_{2}+b_{l})\\ \vdots \\ g(w_{1}\cdot x_{Q}+b_{1})g(w_{2}\cdot x_{Q}+b_{2})\ldots g(w_{l}\cdot x_{Q}+b_{l}) \end{array}\right]}_{Q\times l}$$

When training samples, if the number of neurons in the hidden layer is equal to the number of training set samples, then for any *w* and *b*, to minimize the output error, then:

(21) $$\sum_{j=1}^{Q}\left\|t_{j}-y_{j}\right\|=0$$

where $y_{j}={\left[y_{1j},y_{2j},\ldots ,y_{mj}\right]}^{T}(j=1,2,\ldots ,Q)$ .

When the number of samples *Q* in the training set is large, in order to reduce the computational load, the number *K* of neurons in the hidden layer is usually taken as a smaller number than *Q*, and the training error can approach any ε>0 , that is,

(22) $$\sum_{j=1}^{Q}\left\|t_{j}-y_{j}\right\|<\varepsilon$$

When the activation function g(x) is infinitely differentiable, the parameters of ELM do not all need to be adjusted. *w* and *b* can be randomly selected before training and remain unchanged during the training process. The connection weight μ between the hidden layer and the output layer can be obtained through Equation (23):

(23) $$\underset{\beta }{\min}\left\|H\mu -T^{\prime }\right\|$$

Its solution is

(24) $$\hat{\mu }=\tilde{H}T^{\prime }$$

where $\tilde{H}$ is the Moore–Penrose generalized inverse of the output matrix *H* of the hidden layer.

## 3. Fault Diagnosis Methods

Gearboxes are critical components in rotating mechanical equipment, and planetary gearboxes often operate at high speeds. Overloading under harsh conditions such as heavy loads and non-stable operation leads to excessive force on the gears, causing them to break and having a major influence on the transmission system, which in turn results in the failure of mechanical devices. To enhance the reliability of gearboxes and guide maintenance, conducting research on the faults of planetary gearboxes with broken teeth will provide theoretical support for analyzing the degree of tooth breakage in planetary gearboxes and enabling accurate diagnosis to identify the degree of tooth fracture.

The multi-scale wavelet packet energy entropy method combines the multi-scale wavelet packet energy entropy feature extraction method with the ELM (MSWPEE-ELM) diagnostic model. The fault diagnosis method of MSWPEE-ELM proposed in this paper is divided into two stages: The feature extraction stage, based on the energy entropy of multi-scale wavelet packets, and the classification stage, combining the ELM model and the k-fold cross-validation method. In the fault diagnosis of broken teeth, the characteristics of the signal in the frequency domain and time domain are analyzed more comprehensively, and the uniformity of the energy distribution of the system is evaluated. This method takes into account the issue of the energy entropy value of the wavelet packet formed by the node signal of a specific frequency band under different working conditions. The feature vectors derived from multi-scale information can effectively capture fault information. The combination of the ELM model and the k-fold cross-validation method improves the accuracy of model evaluation. The combination of multi-scale wavelet packet energy entropy and the extreme learning machine fault diagnosis method has filled the gap in the research on the severity of gearbox faults. The feasibility and effectiveness of this method were verified through the comparison of experimental results. These stages are described in the following sections.

### 3.1. Fault Diagnosis Method Process

The specific steps of the multi-scale wavelet packet energy entropy and ELM fault diagnosis method proposed in this paper are as follows:

Step 1: Collect the experimental vibration signals under different fault conditions.

Step 2: Perform multi-scale division processing on the collected experimental vibration signals to decompose the original signals into three sub-signals of different scales. Continue decomposing the high-frequency and low-frequency sub-bands until the desired scale and frequency resolution are reached. Subsequently, reconstruct the obtained wavelet coefficients to approximate the original signal.

Step 3: Calculate the wavelet packet energy entropy of each node under different scale factors and under different operating conditions after reconstruction; the obtained wavelet packet energy entropy of each node under different scale factors is used to construct the feature vector of the signal.

Step 4: By calculating the energy entropy to construct feature vectors, and through cross-validation iterative testing, adjust the number of nodes in the hidden layer and test different types of functions to select the optimal excitation function.

Step 5: Input the fault feature vectors into the ELM model for classification and diagnosis. Identify the fault information and use the ten-fold cross-validation method to improve the accuracy of the evaluation.

**Figure 2:**
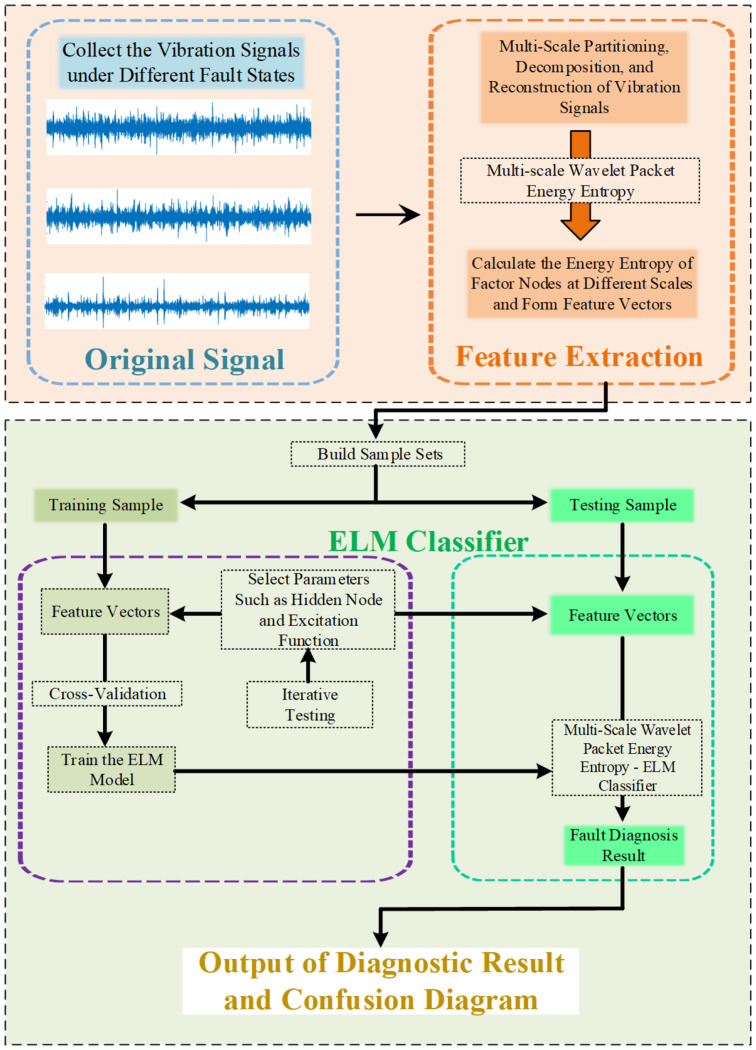
Flow chart of fault diagnosis.

The specific process of fault diagnosis is shown in Figure 2.

### 3.2. Experimental Setup

**Figure 3:**
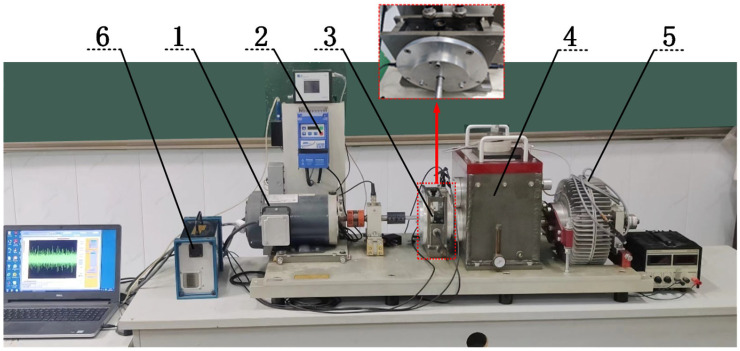
Planetary gear experimental bench: 1—Drive motor; 2—Driver controller; 3—Planetary gearbox; 4—Parallel shaft gearbox; 5—Magnetic powder brake; 6—Data collector.

The comprehensive experimental bench for the planetary gear train, designed for gear transmission fault diagnosis with gear damage, is shown in Figure 3. The red box indicates the sensor mounting location used for collecting the experimental signals.

**Figure 4:**
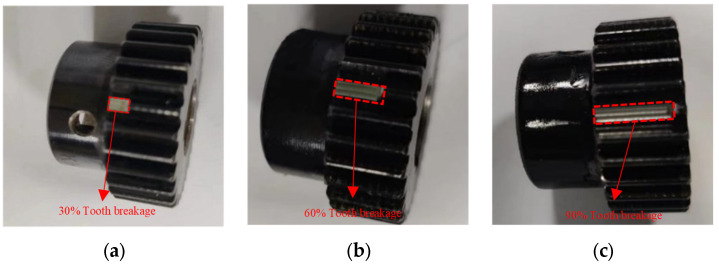
Physical picture of the sun gear with broken tooth fault. Degree of tooth breakage: (**a**) φ = 30%, (**b**) φ = 60%, (**c**) φ = 90%.

The input rotational frequency of the controller is set via the computer, and the rotational speed is allowed to stabilize. Load command is applied to the magnetic powder brake, and power is transmitted to the planetary gearbox through a coupling. This experimental bench is capable of adjusting both speed and torque and can simulate common gear faults. The sun gear has 28 teeth, and the planet gear has 36 teeth. There are 4 planet gears, and the ring gear has 100 teeth. To confirm the accuracy of the simulated physically faulty sun gear, Figure 4a–c show the physical diagrams with the fracture degrees of the sun gear teeth at 30%, 60%, and 90%, respectively. The broken tooth condition of these sun gears belongs to brittle failure. If the length of the broken tooth is X and the maximum possible fracture length is Y , then the degree of tooth breakage φ can be defined:

(25) $$\varphi =\frac{X}{Y}\times 100\%$$

The design of the laboratory bench is conducive to the installation of various sensors. Common acceleration sensors can be installed on gearboxes and bearing housings to measure vibration signals in the horizontal and vertical directions. The data acquisition software and hardware system can be used for vibration signal acquisition and time-domain and frequency-domain analysis of the collected signals. Before conducting the vibration test, the planetary gearbox should be preheated without load for 30 min. To protect the test bench, the input shaft speed should not exceed a certain limit in the presence of gear faults. The input shaft speed is set to 1500 r/s, corresponding to an input rotational frequency *f* of 25 Hz, and the torque of the planetary carrier is set to 5 N·m. The maximum analysis frequency is set to 2000 Hz, and the sampling frequency is 2.56 times that of the maximum analysis frequency, with a sampling duration of 1.6 s.

## 4. Experimental Simulation and Data Analysis

Following the multi-scale wavelet packet energy entropy approach for gear fault feature extraction introduced in Section 2, it aims to solve the problem that the traditional single-scale wavelet packet energy entropy method has certain limitations in extracting fault features and improving diagnostic accuracy. This method conducts multi-scale analysis of the original signal, extracts the wavelet packet energy entropy feature at each scale, and forms the feature vector considering multi-scale information. Then, ELM machine learning classification models are used to classify and diagnose the fault feature vectors, and comparative experiments are conducted with the fault diagnosis methods based on the energy entropy of the single-scale wavelet packet and the traditional entropy value (approximate entropy is taken as an example in this paper).

### 4.1. Fault Feature Extraction

#### 4.1.1. Experimental Signal Acquisition and Analysis

**Figure 5:**
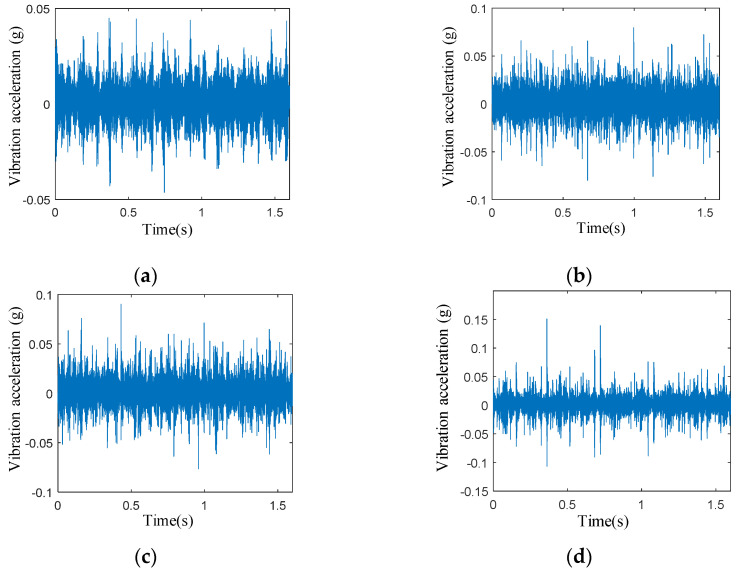
Time-domain vibration signal of the system: (**a**) healthy condition, (**b**) 30% tooth breakage damage condition, (**c**) 60% tooth breakage damage condition, (**d**) 90% tooth breakage damage condition.

To verify the accuracy and efficiency of the MSWPEE-ELM fault diagnosis method proposed in this paper, the vibration acceleration signals of the sun gear of the planetary gearbox under four different degrees of tooth breakage, namely normal, 30% broken, 60% broken, and 90% broken, were collected by using the planetary gear experimental bench mentioned in Section 3.2. Figure 5a–d show the measured vibration acceleration signals of the planetary gearbox transmission system.

By examining Figure 5a–d, it is evident that under healthy gear conditions, the vibration acceleration remains within the range of [−0.05 g, 0.05 g]. However, when a tooth breakage fault occurs, both the maximum amplitude and fluctuation range of the vibration signal on the box surface increase significantly. Additionally, as the extent of the tooth breakage worsens, the impact of the vibration signal intensifies. When the degree of tooth breakage in the sun gear ranges from 0% to 30%, the increase in both the maximum amplitude and fluctuation range of the vibration signal is minimal. In contrast, when the degree of tooth breakage exceeds 30%, both the maximum amplitude and fluctuation range increase substantially as the degree of tooth breakage rises.

Table 1 presents data that clearly indicate the increasing extent of tooth breakage; the maximum amplitude of the vibration signal on the box surface in the time domain increases significantly, with the amplification rate also progressively rising. This suggests that varying degrees of sun gear tooth breakage faults lead to differentiated vibration responses within the system. The findings also show that, across different degrees of tooth breakage, there are significant changes in the vibration characteristics of the planetary gearbox transmission system. By analyzing these changes, the degree of fault in the sun gear’s broken teeth can be more accurately detected and identified.

**Figure 6:**
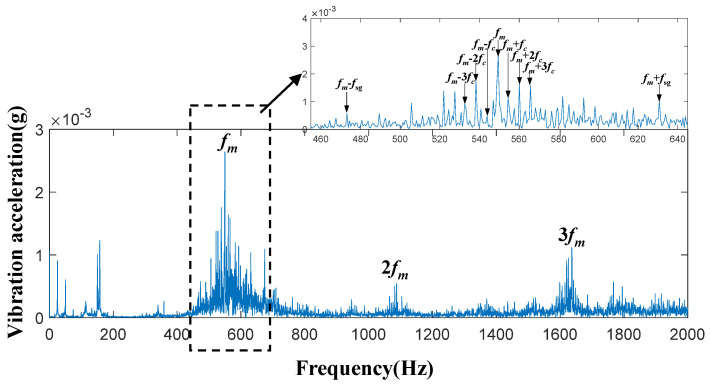
System frequency-domain vibration signals under the healthy condition.

**Figure 7:**
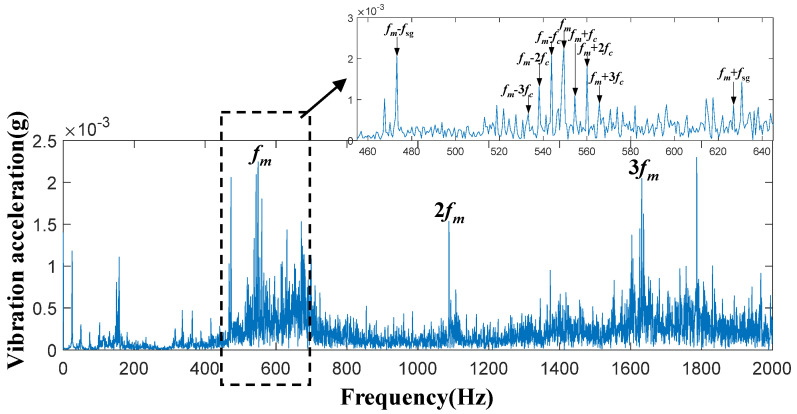
System frequency-domain vibration signals under the 30% tooth breakage damage condition.

**Figure 8:**
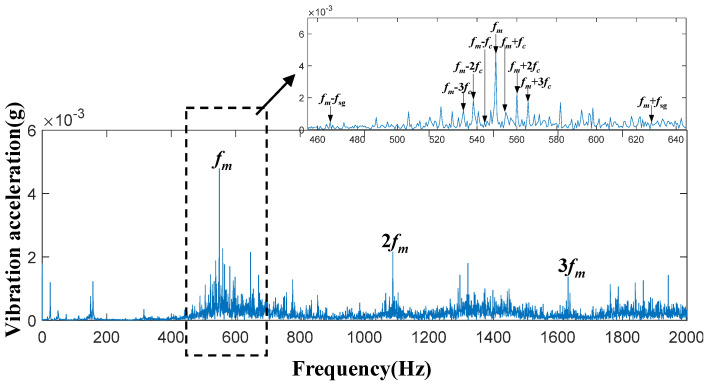
System frequency-domain vibration signal under the 60% tooth breakage damage condition.

**Figure 9:**
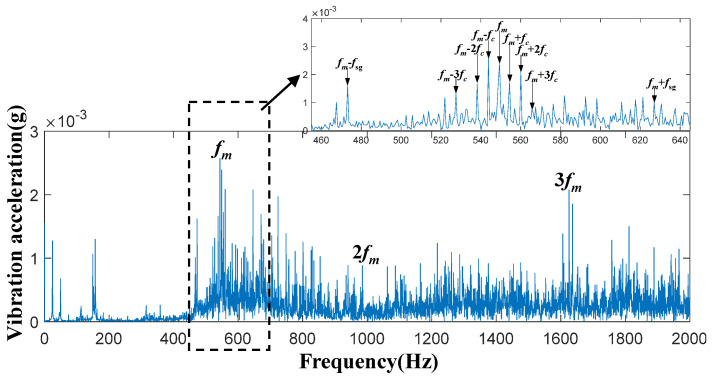
System frequency-domain vibration signals under the 90% tooth breakage damage condition.

In this planetary gearbox transmission system, time-domain acceleration signals of the sun gear were collected under four conditions: the healthy state, 30% tooth breakage, 60% tooth breakage, and 90% tooth breakage. Spectral analysis was then performed on these signals. Figure 6, Figure 7, Figure 8 and Figure 9 present the spectral diagrams and magnified images of the vibration acceleration signals on the box surface. In these figures, the black boxes highlight partial magnifications of the meshing frequency and its sideband spectrum for each condition.

By observing Figure 6, Figure 7, Figure 8 and Figure 9, it can be seen that significant peaks exist at the gear meshing frequency $f_{m}$ and its harmonics $2\;f_{m}$ and $3\;f_{m}$ . Additionally, sideband frequencies around the meshing frequency contain $f_{c}$ and $f_{sg}$ . The specific calculation equations is as follows:

(26) $$f_{c}=\frac{f}{1+\frac{Z_{r}}{Z_{s}}}$$

(27) $$f_{sg}=N\left(\frac{f_{m}}{Z_{s}}\right)$$

where $f_{c}$ denotes the planet carrier rotation frequency, *f* represents the rotational frequency of the sun gear, $Z_{r}$ is the internal gear ring gear, $Z_{s}$ represents the number of teeth on the sun gear, N represents the number of planetary gears, and $f_{sg}$ represents *N* times the sun gear fault characteristic frequency.

It should be noted that even in a healthy state, the frequency of faults can still occur, and the main reason for this lies in the inevitable gear processing errors. Furthermore, the degree of broken tooth damage cannot be judged simply by the vibration amplitude at frequency $f_{m}\pm f_{sg}$ . Since directly diagnosing the severity of sun gear tooth breakage from the spectrum is relatively difficult, developing a more efficient identification method is of great importance.

#### 4.1.2. Multi-Scale Division of Experimental Signals

**Figure 10:**
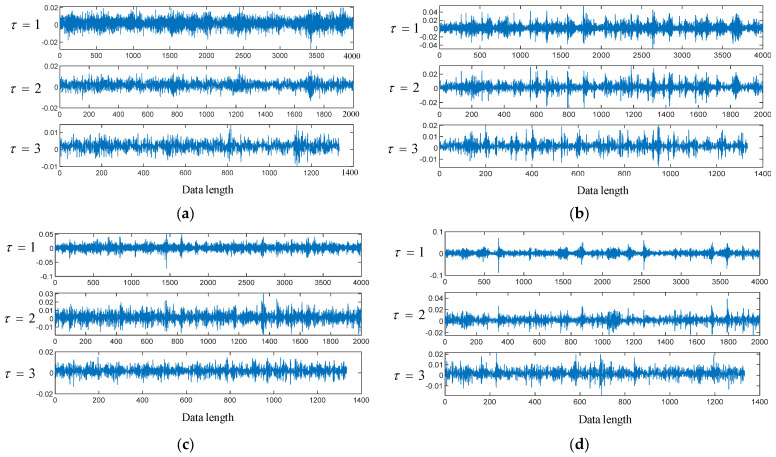
Sub-signals of different scale factors under different working conditions: (**a**) Normal operating condition, (**b**) 30% tooth breakage, (**c**) 60% tooth breakage, (**d**) 90% tooth breakage.

According to the dynamic characteristic experimental tests of the aforementioned planetary transmission system, to perform multi-scale decomposition of the vibration signals collected under various operating conditions in the experiment, 4000 sampling points were selected, and non-overlapping data segments were adopted for each vibration signal. The signals were then decomposed into three sub-signals with different scale factors using Equation (7). Figure 10a–d show the sub-signals corresponding to the different scale factors under different operating conditions.

#### 4.1.3. Wavelet Packet Decomposition of the Experimental Signal

**Figure 11:**
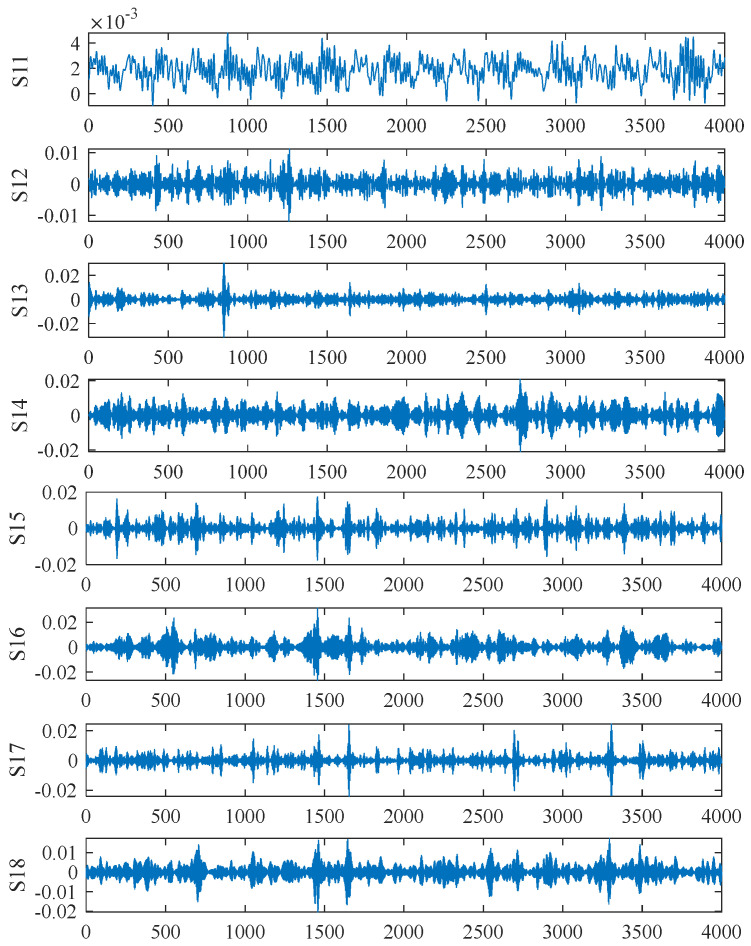
Wavelet coefficient diagram under 30% tooth breakage operating condition.

To obtain feature vectors from the gearbox’s vibration acceleration signals, this section applies multi-scale wavelet packet decomposition to the acceleration signals under normal conditions as well as under 30%, 60%, and 90% tooth breakage severities. Given the non-stationary nature of the experimental signals and the potential for noise interference, the dmey wavelet function is employed for decomposition. The raw signals are first divided into sub-signals using three scale factors. Each sub-signal is then decomposed into three layers within the wavelet packet, with adjacent sub-bands averaged at each layer, ultimately yielding eight sub-bands. By reconstructing these sub-bands, the wavelet coefficient plots for the different operating conditions are obtained. Only the wavelet coefficient plots for the 30% tooth breakage signal are presented in this paper. Figure 11 illustrates the distribution of wavelet coefficients at each level for the 30% tooth breakage signal.

**Figure 12:**
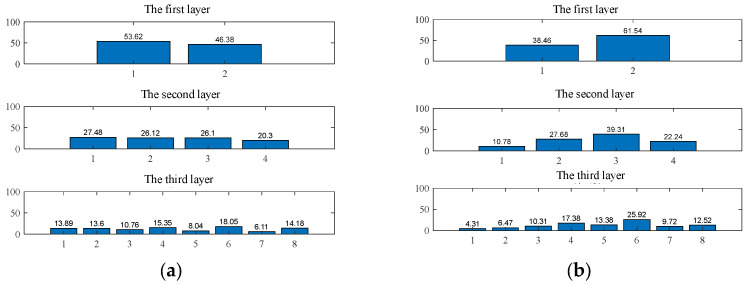

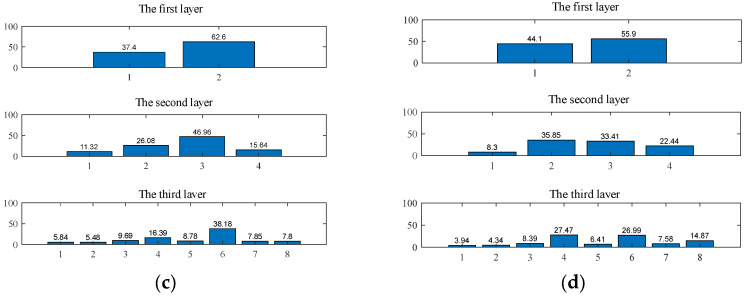
Wavelet packet-based node energy distribution across decomposition layers under multiple operating conditions: (**a**) Normal operating condition, (**b**) 30% tooth breakage, (**c**) 60% tooth breakage, (**d**) 90% tooth breakage.

After wavelet packet decomposition, the energy of the original signal was distributed across the sub-frequency bands. The energy ratio of the reconstructed time-domain signals to the total energy was calculated using Equations (9) and (10), from which the multi-scale wavelet packet energy entropy at each node of the sub-frequency bands was extracted. Figure 12 illustrates the energy distribution at each node of each layer under different operating conditions when the scale factor is set to 1.

#### 4.1.4. Feature Vector Calculation

According to Equation (11), the wavelet packet energy entropy values of specific nodes in a single frequency band under different working conditions are calculated, respectively, when the scale factor is equal to 1, 2, and 3. Table 2 shows the wavelet packet energy entropy values of each node under different scale factors.

Traditional methods that use wavelet packets to diagnose gear faults typically focus on the signal complexity at a single scale, neglecting the comprehensive detection and extraction of local feature information from the experimental vibration signal. To address this limitation, Table 2 presents the wavelet packet energy entropy values for each node after decomposition and displays the wavelet packet energy entropy values for each node when the scale factors are 1, 2, and 3, respectively. This allows for a more comprehensive evaluation of the signal’s local characteristics from a multi-scale perspective. Compared with traditional methods that consider only a single scale, the use of multi-scale features provides a more accurate representation of the signal’s information, consequently increasing the accuracy of fault diagnosis. In contrast, Table 3 displays feature vectors obtained through the traditional extraction method, which considers only the features of a single scale and ignores information from other scales.

From Table 2, it can be observed that under identical operating conditions, as the scale factor increases, the wavelet packet energy entropy values of sub-bands S11 and S18 gradually rise, whereas those of sub-bands S12 and S13 exhibit a progressive decline. When the scale factor is set to 1, the entropy values of sub-bands S12 and S14 decrease steadily with increasing tooth breakage severity. At a scale factor of 2, the entropy value of sub-band S14 diminishes with higher breakage severity, while sub-band S16 shows an upward trend. Finally, at scale factor 3, the entropy values of sub-bands S11, S12, S14, and S16 all demonstrate a consistent reduction as tooth breakage severity escalates.

### 4.2. Fault Diagnosis and Comparison Verification

The widespread application of machine learning technology in the field of fault diagnosis has transformed the traditional, manually dependent fault diagnosis method into intelligent diagnosis, significantly improving the accuracy of mechanical fault detection and greatly reducing the computing time. In classification diagnosis tasks, algorithms such as NBC, KNN, and fuzzy logic are widely used. However, these methods have high computational costs when training large-scale datasets, rely on manually setting a large number of hyperparameter tuning models, and the training process usually takes a long time. This section studies ELM as the mechanical fault classification and diagnosis technology and demonstrates the practicability of this method in the classification of gear tooth breakage degree through experimental data.

#### 4.2.1. ELM Parameter Selection

The extreme learning machine (ELM) was utilized as the classifier to classify and diagnose the fault feature vectors derived from the horizontal and vertical vibration signals of the planetary gearbox. Prior to classification, the number of nodes in the hidden layer was adjusted through continuous iterative testing, and the optimal number of nodes that maximizes classification accuracy was selected. Additionally, various excitation functions were tested to identify the optimal function that significantly enhanced the classification performance.

This section utilized multi-scale wavelet packet energy entropy decomposition to decompose the original signal into multi-scale components followed by the calculation of energy entropy values for each component, which serve as fault feature vectors. A total of 320 samples were used, with 160 randomly selected feature vector matrices assigned to the training set and the remaining 160 to the test set. Energy entropy was computed from the experimental data to form feature vectors, and appropriate hidden nodes and activation functions were selected.

**Figure 13:**
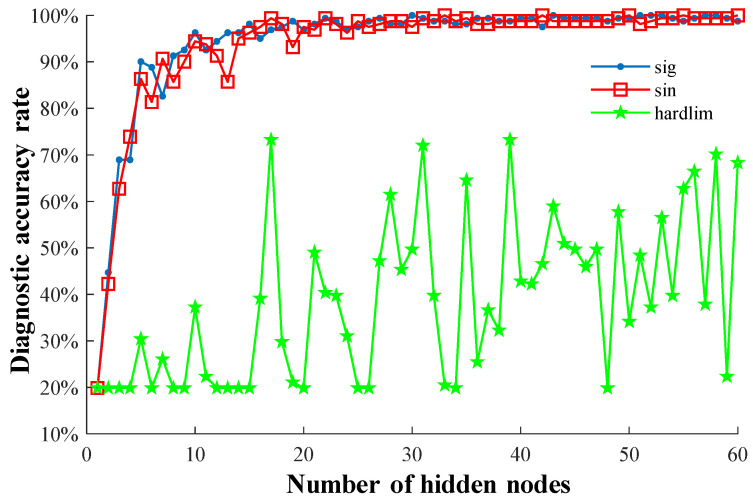
Comparison of common excitation functions.

After many repeated experiments, the number of nodes in the hidden layer was 18; with the excitation function as the sig function, the classification accuracy rate could reach a high value. After that, increasing the quantity of nodes in the hidden layer made no significant change to the classification accuracy rate. Therefore, the number of hidden layer nodes of the ELM model in this study was set at 18. This setting can optimize the classification influence of the model on the test data. Further, by comparing three excitation functions (sig, sin, and hardlim), data classification of gear tooth breakage faults was performed using the ELM model. According to the results shown in Figure 13, when the excitation function was chosen as sig, the test accuracy of the ELM model outperformed that of the other two excitation functions. Therefore, the sig excitation function was used in the ELM model.

#### 4.2.2. Fault Identification

This section performs fault diagnosis on the sun gear under four different tooth breakage severity levels: normal condition, 30% tooth breakage, 60% tooth breakage, and 90% tooth breakage, in accordance with the fault classification methodology outlined in this study.

**Figure 14:**
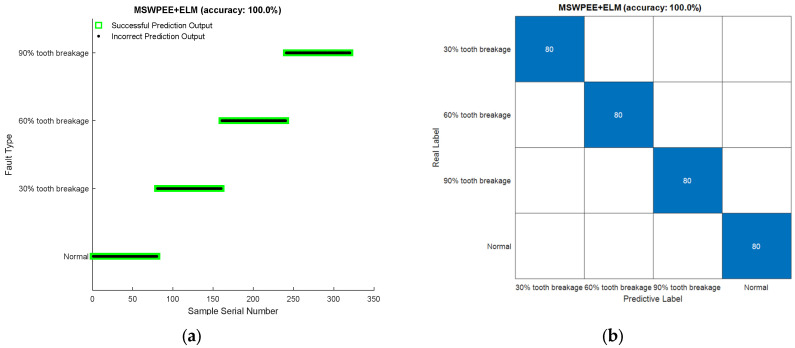
Intelligent classification results of sun gear tooth breakage degree by ELM: (**a**) fault diagnosis classification results, (**b**) fault diagnosis confusion diagram.

According to Section 4.1, the original signal was subjected to multi-scale partitioning through wavelet packet decomposition. The wavelet packet algorithm split the signal into three layers at different scale factors, resulting in eight sub-bands, followed by the reconstruction of the sub-band signals. Next, the wavelet packet energy entropy for specific nodes in each frequency band was calculated under varying operating conditions. These entropy values were aggregated to form a feature vector matrix, which quantitatively characterized the information patterns within the raw vibration signals. The characteristic vectors of the four different fracture states of the sun gear were integrated into a dataset. The dataset contained a total of 320 samples (40 samples for each fault type). Using the feature vector matrices extracted under different tooth fracture conditions as the input, ten-fold cross-validation was employed to enhance the reliability of the model. The fault classification result and confusion matrices for the ELM model are presented in Figure 14.

According to Figure 14, the ELM classifier was applied to the classification of the broken tooth state of the sun gear in the planetary gearboxes. The fault diagnosis accuracy rate obtained was 100%. The experimental results verified the effectiveness and feasibility of the MSWPEE-ELM method proposed in this study in the feature extraction and fault diagnosis of the broken tooth state of the sun gears.

### 4.3. Comparison of Different Methods

**Figure 15:**
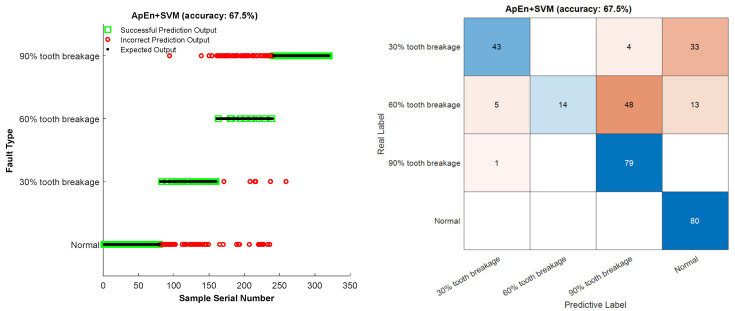
ApEn-SVM fault diagnosis method.

**Figure 16:**
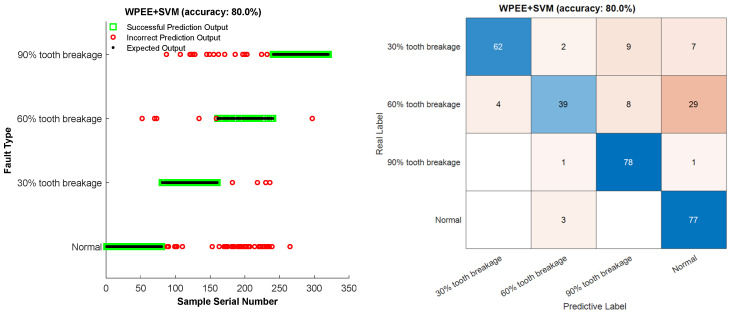
WPEE-SVM fault diagnosis method.

**Figure 17:**
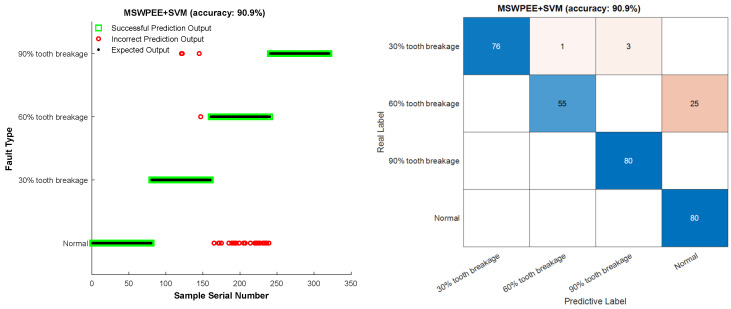
MSWPEE-SVM fault diagnosis method.

**Figure 18:**
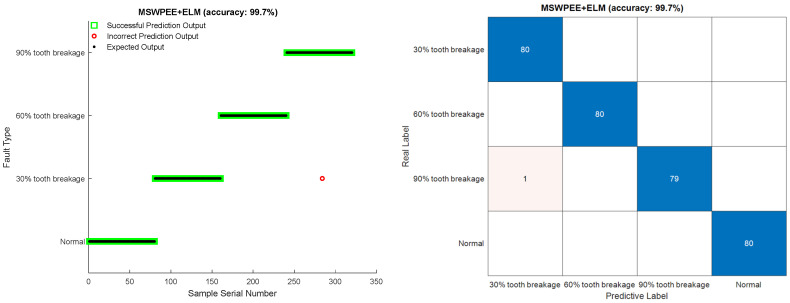
MSWPEE-ELM fault diagnosis method.

To verify the superiority and universality of the method proposed in this study, comparative experiments were conducted by combining other feature extraction methods with different machine learning classifiers. Method 1: approximate entropy (ApEn)-SVM, Method 2: traditional wavelet packet energy entropy (WPEE)-SVM, Method 3: MSWPEE-SVM, Method 4: MSWPEE-ELM. To more effectively demonstrate the universality of the tooth breakage degree fault diagnosis method proposed in this paper, Gaussian white noise was added to the collected dataset to make it closer to the actual working conditions of planetary gearboxes in engineering applications. Four methods were used to extract features and identify faults on the signals. The fault diagnosis results are shown in Figure 15, Figure 16, Figure 17 and Figure 18.

As shown in Figure 15, the APEN-SVM fault diagnosis results indicate that the recognition accuracy of this method is 67.5%. From the experimental output results, it can be seen that this method can effectively distinguish whether the gear has a broken tooth fault, but the effect is poor in diagnosing and identifying the type of broken tooth degree. This is because the approximate entropy can help identify abnormal changes in the gear system. Because it focuses on the structural features of the signal rather than merely numerical fluctuations, it performs relatively robust-free when dealing with noisy signals. This enables it to perform well in actual gear vibration signals and effectively distinguish between normal and abnormal vibration states. However, it mainly focuses on the complexity of the signal rather than specific frequency components or amplitude variations. As a result, it cannot clearly indicate the specific type of fault by itself. In this study, the research direction of diagnosing and identifying faults with different degrees of gear tooth breakage is less effective.

Due to the fact that different degrees of broken teeth can lead to different patterns of vibration signal fluctuations, each type of fault usually occurs in a specific frequency band, and each fault type has distinct characteristic frequency bands. Energy entropy, by quantifying the energy distribution of the signal to reveal signal fluctuations, can better capture the spectral changes caused by broken teeth. The variation in different degrees of broken teeth is reflected by the change in the degree of energy concentration. By combining the wavelet packet theory to form the wavelet packet energy entropy, the energy distribution of the signal in different frequency bands is measured. The features of the signal are extracted by calculating the uncertainty of the signal’s energy distribution. As shown in the diagnostic results of Figure 16, the fault recognition accuracy rate of WPEE-SVM is 80%.

The analysis of the experimental results shows that when WPEE diagnoses gear faults, it considers the complexity of the signal at a single scale only and fails to comprehensively detect and extract the local characteristic information in the experimental vibration signal. On this basis, the coarse-grained theory is introduced to evaluate the local characteristics of the signal more comprehensively from a multi-scale perspective, better reflecting the information of the signal itself, which is conducive to improving the accuracy of fault diagnosis. As shown in the result of Figure 17, the recognition accuracy of MSWPEE-SVM is 90.9%, which is 10.9% higher than that of WPEE-SVM in diagnosing the same gear vibration signal. Through experimental comparison, it is verified that MSWPEE has certain advantages in the vibration signal features of gears with different degrees of broken teeth.

As shown in Figure 18, for the proposed method MSWPEE with ELM classifier, the accuracy rate of fault diagnosis and recognition for different degrees of broken teeth of the sun gear has reached 99.7%. The experiment demonstrates the efficiency and accuracy of the multi-scale wavelet packet energy entropy feature extraction method. Due to the unique closed-form solution of the extreme learning machine (ELM) model, it effectively mitigates overfitting. Moreover, without the need to train the hidden layer network parameters, it can fully leverage its representational capacity to capture the true distribution of the samples. The parameters of the hidden layer generate an optimal decision boundary, offering greater stability and generalization ability. The fault diagnosis results further validate the efficiency of the ELM model.

To evaluate the reliability and efficiency of the experiment while avoiding deviations, the experiment was repeated 10 times. The average recognition accuracy rate was taken, and the fault identification time was introduced for comparison. The results are shown in Table 4.

It can be seen from the table that the results of the repeated experiments are roughly similar to those in Figure 15, Figure 16, Figure 17 and Figure 18. The fault recognition accuracy of ApEn-SVM is 67.94%, and the average recognition accuracy of the feature extraction method based on energy entropy has reached more than 80%. The average recognition accuracy of the fault diagnosis method proposed in this study can reach more than 99%, achieving satisfactory results. And it has a significant advantage in terms of fault identification time. Comprehensive analysis shows that the MSWPEE-ELM fault diagnosis method proposed in this study is highly efficient and versatile in the classification and identification of broken teeth of different degrees. It can be applied to solve practical engineering problems and has a good application prospect.

## 5. Conclusions

This paper presents a fault diagnosis method for broken teeth in solar gears based on multi-scale wavelet packet energy entropy and extreme learning machine (MSWPEE-ELM). This method conducts multi-scale analysis of the original signal, extracts the wavelet packet energy entropy features at each scale, forms a feature vector considering multi-scale information, and then classifies and diagnoses the faults through the ELM classifier. The experimental results show that multi-scale analysis can describe the fault information in the signal more comprehensively. Through comparative experiments, it is superior to other algorithms in the feature extraction of solar gear tooth breakage faults and the classification and diagnosis of tooth breakage degree, and it has better fault diagnosis accuracy and good application prospects. Subsequent research can also be extended to the analysis of other fault types and multi-gear faults, and the fault diagnosis model can be optimized to improve the accuracy of fault classification and diagnosis efficiency.

## Figures and Tables

**Table 1:** Maximum amplitude of vibration for the tooth breakage degree φ .

φ(%)	0	30	60	90
Maximum amplitude of vibration (g)	0.04486	0.0795	0.09027	0.1511

**Table 2:** Partial feature vector data obtained from the fault feature extraction method based on multi-scale wavelet packet energy entropy.

Gear Operating Condition	Scale Factor	S11	S12	S13	S14	S15	S16	S17	S18
Normal	1	0.27	0.27	0.28	0.23	0.17	0.27	0.30	0.20
	2	0.34	0.12	0.15	0.31	0.26	0.31	0.25	0.15
	3	0.36	0.05	0.13	0.14	0.17	0.35	0.23	0.27
30% tooth breakage	1	0.13	0.17	0.30	0.23	0.22	0.26	0.34	0.26
	2	0.23	0.06	0.18	0.24	0.32	0.33	0.26	0.27
	3	0.30	0.04	0.07	0.16	0.24	0.32	0.28	0.35
60% tooth breakage	1	0.16	0.15	0.29	0.22	0.19	0.19	0.36	0.21
	2	0.24	0.13	0.23	0.21	0.31	0.33	0.28	0.24
	3	0.30	0.04	0.21	0.19	0.25	0.30	0.26	0.33
90% tooth breakage	1	0.12	0.13	0.35	0.20	0.19	0.28	0.35	0.17
	2	0.20	0.04	0.14	0.20	0.33	0.36	0.24	0.21
	3	0.28	0.04	0.08	0.14	0.21	0.24	0.29	0.36

**Table 3:** Feature vector data obtained via the traditional wavelet packet energy entropy with a single scale.

Gear Operating Condition	S11	S12	S13	S14	S15	S16	S17	S18
Normal	0.27	0.27	0.28	0.23	0.17	0.27	0.30	0.20
30% tooth breakage	0.13	0.17	0.30	0.23	0.22	0.26	0.34	0.26
60% tooth breakage	0.16	0.15	0.29	0.22	0.19	0.19	0.36	0.21
90% tooth breakage	0.12	0.13	0.35	0.20	0.19	0.28	0.35	0.17

**Table 4:** The accuracy and identification time for different fault identification algorithms.

Methods	Accuracy of Correctly Identified Samples (%)				Fault Identification Time(s)	Average Accuracy (%)
	Category 1	Category 2	Category 3	Category 4		
ApEn-SVM	98.875	52	78	98.875	0.51165	67.94
WPEE-SVM	95.625	79.5	49.375	97.5	0.51711	80.25
MSWPEE-SVM	100	86.125	72.875	100	0.54475	89.75
MSWPEE-ELM	100	98.5	99.125	99.875	0.02237	99.38

## Data Availability

The data that support the findings of this study are available upon request from the authors.
